# Bicycle Helmet Wearing Is Not Associated with Close Motor Vehicle Passing: A Re-Analysis of Walker, 2007

**DOI:** 10.1371/journal.pone.0075424

**Published:** 2013-09-25

**Authors:** Jake Olivier, Scott R. Walter

**Affiliations:** 1 School of Mathematics and Statistics, University of New South Wales, Sydney, Australia; 2 Centre for Health Systems and Safety Research, Australian Institute of Health Innovation, University of New South Wales, Sydney, Australia; University of Oxford, United Kingdom

## Abstract

**Objectives:**

To re-analyse bicycle overtaking data collected by Walker (2007) with a view to assess factors associated with close passing (<1 m), to adjust for other observed factors in a multivariable analysis, and to assess the extent to which the sample size in the original analysis may have contributed to spurious results.

**Method:**

A re-analysis of 2,355 motor vehicle passing events recorded by Walker that includes information on cyclist's distance to the kerb, vehicle size and colour, city of observation, time of day, whether the event occurred while in a bikelane and helmet wearing. Each variable was considered for a final, multivariable model using purposeful selection of variables. The analysis was repeated using multiple logistic regression with passing distance dichotomised by the one metre rule. Bootstrap p-values were computed using sample sizes computed from conventional values of power and effect size.

**Results:**

The previously observed significant association between passing distance and helmet wearing was not found when dichotomised by the one metre rule. Other factors were found to be significantly associated with close passing including cyclists' distance to the kerb, vehicle size and city of observation (Salisbury or Bristol, UK). P-values from bootstrap samples indicate the significance of helmet wearing resulted from an overly large sample size.

**Conclusions:**

After re-analysis of Walker's data, helmet wearing is not associated with close motor vehicle passing. The results, however, highlight other more important factors that may inform effective bicycle safety strategies.

## Introduction

Motor vehicle collisions with cyclists travelling in the same direction during passing manoeuvres often result in serious injury to the cyclist [1]. To assess possible causes of these collisions, Walker [2] gathered and analysed data on passing manoeuvres by attaching sensors to his bicycle. He measured, through hidden devices, the passing distance of cars from his bicycle at pre-defined distances from the kerb on various routes. For each event, Walker recorded the passing distance, the vehicle type and colour, whether it occurred while in a bike lane, the city (Salisbury or Bristol, UK), the time of day and whether he was wearing a helmet.

In his paper, Walker [2] noted a statistically significant negative association of passing distance (distance between vehicle and cyclist) with both kerb distance (distance from cyclist to kerb) and helmet wearing. He hypothesised that drivers may modify their passing distance when a cyclist is wearing a helmet because they perceive less risk than when a cyclist is not wearing a helmet. There is evidence of behaviour modification associated with helmet wearing in other studies. These have shown that regular helmet wearers decrease their cycling speed when not wearing a helmet [3], that male cyclists slightly increase speed in low speed areas when wearing a helmet [4], unhelmeted cyclists are more likely to commit a traffic violation [5], [6] and that some drivers believe helmet wearers cycling alone may behave more predictably than non-helmet wearers [7]. Despite Walker's hypothesis, there is no clear evidence helmet wearing leads to an increase in injury risk for the cyclist. This includes risk compensation theory which posits the use of safety equipment leads to riskier behaviour by the user. Also, despite strong evidence in support of bicycle helmet efficacy [6], [8]–[10], and the benefit of mandatory helmet legislation [11]–[14], laypersons have used Walker's findings to justify the removal of mandatory helmet laws [15], [16].

Walker [2] also assessed the effect of several other factors on passing distance, including vehicle type and colour, and the location (Salisbury or Bristol, UK) in which each passing event occurred. Only univariate analyses were performed, so it is not possible to ascertain how each factor affected passing distance when considered adjusted for other factors. In other words, the conclusions drawn in Walker's original paper are based on analyses that did not capitalise on the full potential of the data.

In regards to kerb distance, Walker [2] concluded that drivers follow the same overtaking path regardless of where a bicycle is located, but warned that advising cyclists to ride closer to the edge of the road may not increase safety due to a greater likelihood of encountering obstacles (grates, debris and car doors) when close to the kerb.

Noteworthy, the average passing distances in Walker's study were larger than one metre. When a heavy vehicle overtakes a cyclist, lateral forces increase which, in turn, increases the risk of a cyclist collision [17], [18]. These authors have given minimum overtaking distance recommendations of three feet (91.44 cm) and 1.5 m for heavy vehicles passing at 64 km/h and 100 km/h respectively. A minimum distance of one metre, or similar three feet in the USA, for motor vehicles passing cyclists is often recommended and sometimes legislated [18]–[24]. Given the street types cycled by Walker, the observed passing distances were often beyond the recommended safe distance regardless of helmet wearing or the kerb distance. The original analysis defined near passing using data-driven quartiles for various combinations of helmet usage and kerb distance. There are caveats associated with the use of data-driven quartiles [25] and each quartile in the data was well above one metre (range: 1.17–1.47 m). Hence, Walker's analysis did not consider distances of practical importance in the categorisation of passing distance.

**Figure 1:**
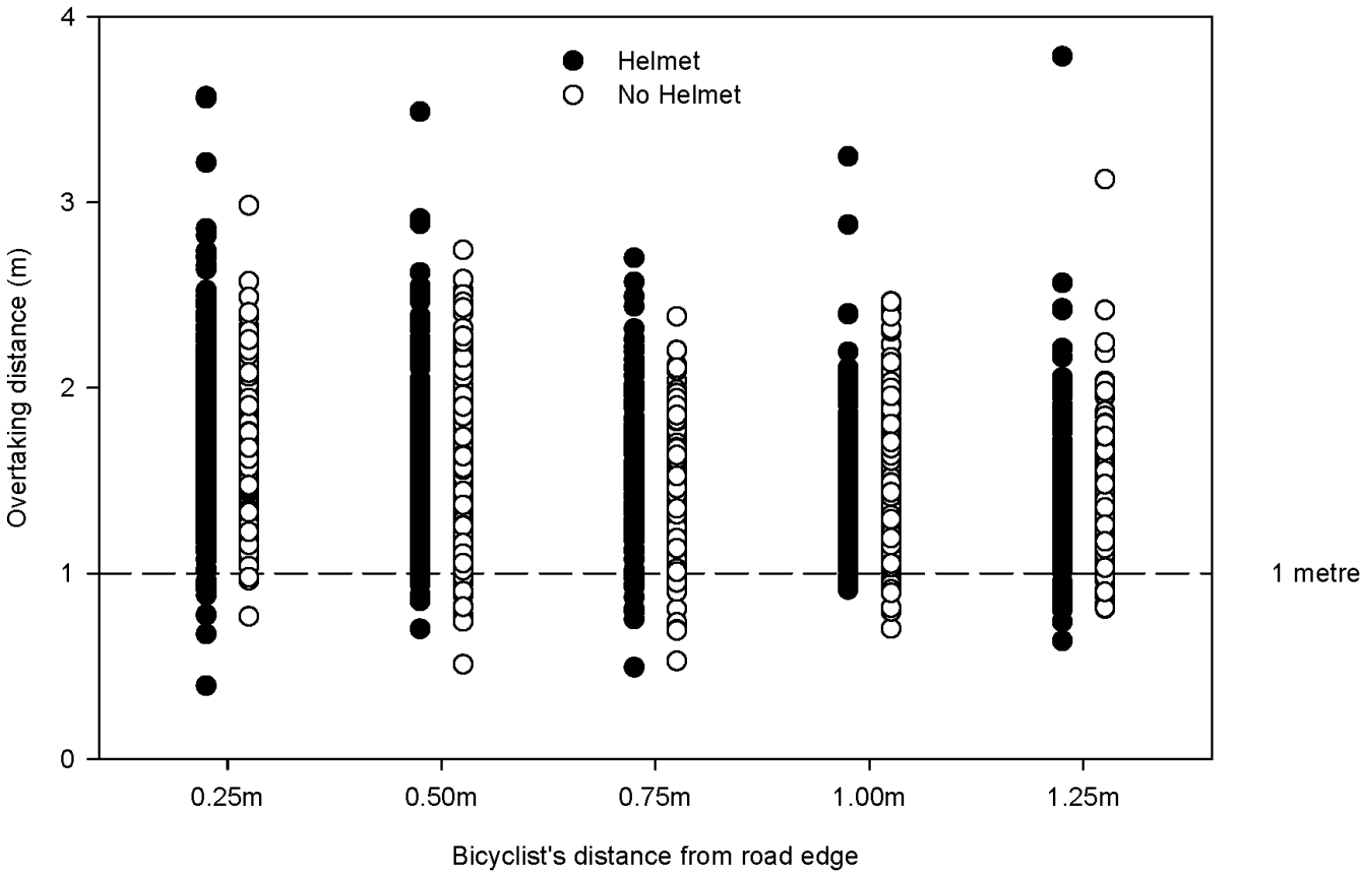
Scatterplot of overtaking distance for helmet wearing and bicyclist's distance from road edge.

In the original study there was 98% power to detect a hypothetical small effect size of *f = 0.1* defined by Cohen [26], associated with the collection of 2355 overtaking events. Whether this power level was calculated *post hoc* or *a priori*, this is an overpowered study far above the usual convention of 80% power being adequate to detect an effect of any size [27] which increases the risk of type I errors, i.e., the detection of a statistically significant difference in the sample when there is no true difference in the population [28]. This has implications for the interpretation of the results reported in the original study. In addition, the power analysis was not based on topic/context specific effect sizes that are more informative than statistical significance resulting from a large sample and small effect size [29]. The smallest effect size that makes an impact on patient outcomes, known as a minimally important difference, should be incorporated in the sample size estimation and reporting of trials when possible [30], [31]. A scatterplot of passing distances in the Walker data set for kerb distance and helmet wearing is given in Figure 1.

Given the potential increase in harm that may arise from not wearing a helmet, it is essential to develop a clear understanding of Walker's data. We aimed to perform a multivariable re-analysis of the original data to determine potential factors associated with motor vehicle passing distances for cyclists. Additionally, we categorised passing distances using the one metre recommendation and assessed potential associations. Lastly, we use a resampling approach to quantify the overall influence of using an excessively large sample size.

## Methods

We downloaded the original raw data set in MS Excel format made available from Walker [32]. Within the Walker data are the predefined variables kerb distance (0.25 m, 0.50 m, 0.75 m, 1.00 m, 1.25 m) and whether a helmet was worn, and the observed variables passing distance (m), time of day, vehicle type and colour categories, city location (Salisbury or Bristol, UK), whether there was a bike lane and street type categories. Walker also reported wearing a wig to give a female appearance; however, this data has not been made available.

For our analysis, we categorised passing distance according to the recommended one metre rule into *close* (less than 1 m) and *far* (greater than or equal to 1 m) distances. We also further combined vehicle type into groups of small (ordinary car, sports utility vehicle/pickup, taxi, powered two-wheelers) and large (light-goods vehicle/minibus, bus, heavy-goods vehicle) sized vehicles. Due to their size and lane restrictions, larger vehicles may give less passing distance than smaller ones. Time of day was categorised to distinguish daily commuting periods (7–10 am, 10 am-2 pm and 2 pm+).

Linear regression was used to assess any association of passing distance as a continuous variable with the remaining study variables. A final multivariable model was chosen using purposeful selection (PS) of variables [33], which has been shown to outperform other model selection procedures at identifying confounding variables [34]. Briefly, the steps of the PS algorithm are to (1) assess each variable individually in a univariate analysis, (2) each variable with a p-value below a nominal threshold (say p<0.15) is put into a multivariable model and variables are removed by backwards elimination, (3) variables eliminated at step (1) are put into the multivariable model and retained by a more stringent criteria (say p<0.1). More liberal p-value cut-offs than the conventional 0.05 is used to identify important, but possibly non-significant variables [34], [35]. Using the binary close/far passing distances as the dependent variable, the PS algorithm was used to develop a multiple logistic regression model. For our analyses, we used p-value cut-offs of p = 0.15 for inclusion in the multivariable analysis at step (2) and p = 0.1 for the final multivariable model at step (3). We report the results from both the univariate and multivariable linear and logistic models.

Although there is strong evidence for a buffer zone between motor vehicles and cyclists, any cut point recommendation is somewhat arbitrary. To further assess the effect the choice of cut point has on our analysis, additional multivariable models were run using various cut points (0.5 m, 0.75 m, 1.5 m and 2.0 m) as a sensitivity analysis.

In addition, we assessed the effect the original study's large sample size has on the significance of helmet wearing for passing distance through a resampling scheme. Since standard errors are decreasing functions of sample size, an inappropriately large sample size can over accentuate the significance of an effect. With that in mind, sample sizes were computed using G*Power for small, medium and large effect sizes [27], [36] and conventional power levels of 80%, 85% and 90% [37] for the analysis of passing distance as a continuous variable and in the binary close/far categorisation. The Walker data was then resampled with replacement for the computed sample size and model coefficients for helmet wearing were estimated for univariate and multivariable models. This process was repeated 200 times to generate a bootstrap sample of coefficients whose standard deviation is an estimate of the standard error for the given sample size [38]. P-values were then computed using the z-test for the ratio of the model coefficient and the bootstrap standard error. The procedure was repeated for kerb distance as a sensitivity analysis.

## Results

To confirm that the downloaded data set was identical to the original study, we reproduced Table 1 in Walker [2] for the numbers of overtaking events for each combination of kerb distance and helmet wearing. We obtained the same frequency of events for each category, but they were swapped for helmet versus no helmet. Through personal communication with Ian Walker, this opposite coding for helmet wearing was verified and did not affect the original analysis (Table 1).

**Table 1:** Number of overtaking events per condition.

	Distance from road edge (m)				
	0.25	0.50	0.75	1.00	1.25
Helmet	244	275	186	272	172
No Helmet	426	270	153	197	160

A preliminary exploratory analysis indicated that city and street type variables were confounded (Table 2). Street types classified as “one-way (two lanes)”, “regular residential street” and “rural” were only observed in Salisbury and the vast majority of events on “main road, regular” occurred in Salisbury (1630/1637≈99.6%). Overtaking events in Bristol were predominantly on “regular urban street” (441/450 = 98%). Due to this confounding, the variable *city* was analysed in lieu of street type.

**Table 2:** Number of overtaking events by city and street type.

	One- way	One- way	Urban	Residential	Main	
	(one lane)	(two lane)	Street	Street	Road	Rural
Salisbury	7	13	214	39	1630	2
Bristol	2	0	441	0	7	0

Additionally, colour categories (blue, red, silver/grey, white, black, green and other) all had similar passing distances, were non-significant for all univariate and multivariable models, and are not reasonably categorised for easier discrimination. *Vehicle colour* has therefore been removed from further consideration. Although the variable *kerb distance* is a continuous measurement, a comparison of univariate models with *kerb distance* as a categorical variable resulted in an improved Akaike information criterion although more degrees of freedom were used to estimate the model. Therefore, the categorical version of *kerb distance* was used for all subsequent analyses. Walker's analysis reported using a square root transformation of passing distance to account for non-normality and the removal of thirty-five atypical observations; however, a normal quantile plot of such a large data set indicates any adjustments were not needed for analyses reliant on an assumption of normality [39].

The results of the linear regression for passing distance as a continuous variable are given in Table 3. The variables *vehicle size*, *time of day*, *kerb distance* and *helmet wearing* were included in an initial multivariable model. *Time of day* was highly non-significant (p = 0.588), so it was removed from the model. The variables *bikelane* and *city* were then included in the model and *city* was retained (p = 0.001) although *bikelane* was not (p = 0.149). The final model estimates a significant, adjusted effect for *vehicle size*, *city*, *kerb distance* and *helmet wearing*. The latter estimates a 5.8 cm average decrease in passing distance while wearing a helmet.

**Table 3:** Univariate and multivariable linear model results for passing distance.

	Univariate			Multivariable		
	Estimate	SE	p- value	Estimate	SE	p- value
Vehicle, Small vs. Large	0.110	0.021	<0.001	0.089	0.020	<0.001
Bikelane, Yes vs. No	0.059	0.055	0.285			
*City	0.028	0.020	0.161	0.064	0.021	0.002
Time of Day						
7–10 AM	−0.104	0.021	<0.001			
10–2 PM	−0.056	0.018	0.002			
2 PM+	(referent)					
Kerb Distance						
0.25 m	0.285	0.025	<0.001	0.281	0.025	<0.001
0.50 m	0.178	0.026	<0.001	0.201	0.027	<0.001
0.75 m	0.093	0.029	0.001	0.114	0.029	<0.001
1.00 m	0.078	0.027	0.003	0.087	0.026	<0.001
1.25 m	(referent)					
Helmet, Yes vs. No	−0.085	0.016	<0.001	−0.058	0.015	<0.001

* City comparison is between Salisbury and Bristol.

**Figure 2:**
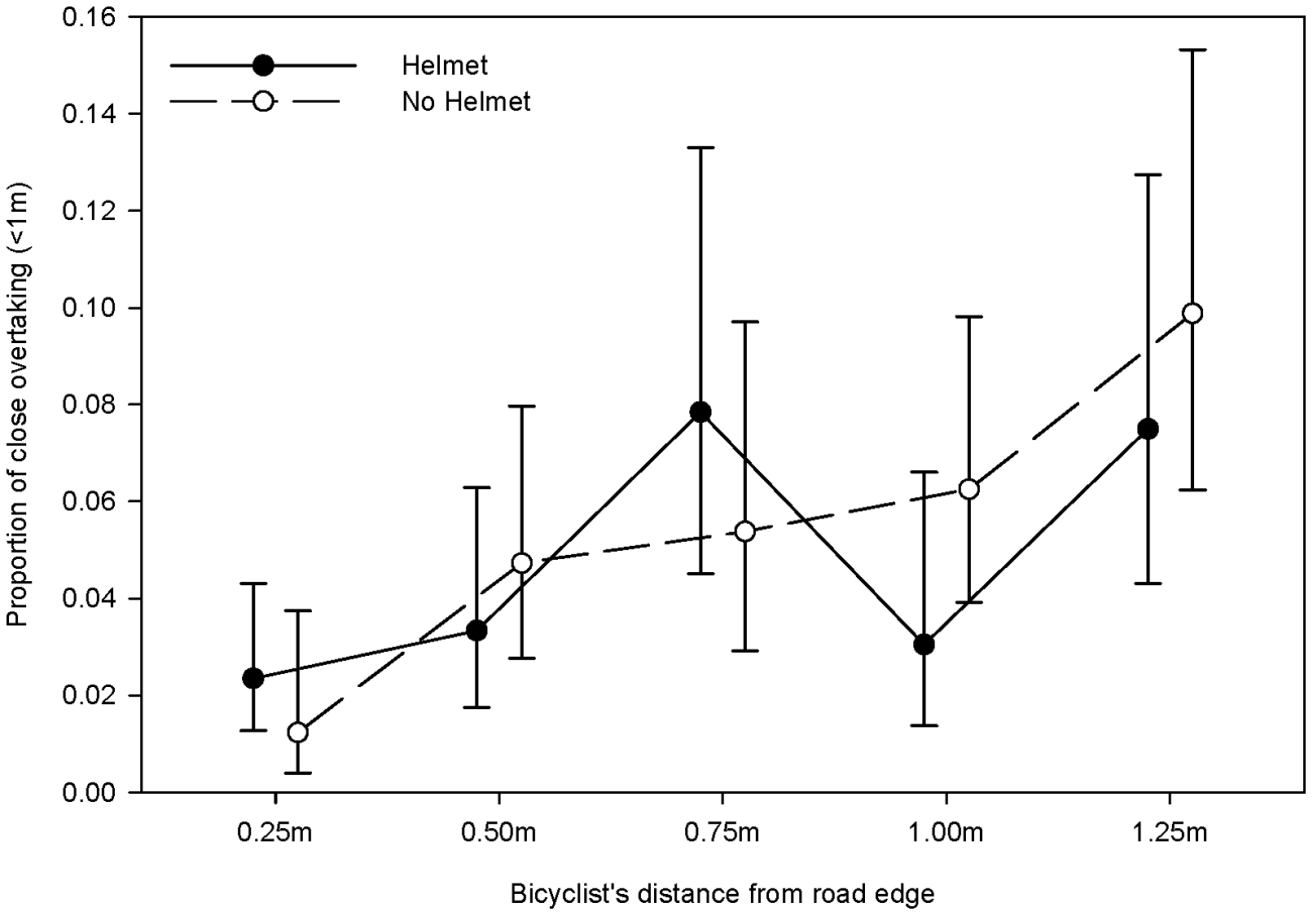
Proportion of close (<1m) overtaking events for helmet wearing and bicyclist's distance from road edge.

Table 4 contains the results from the logistic regression analysis for close and far passing distances. The univariate analyses indicated *vehicle size*, *city* and *kerb distance* were important to include in a multivariable analysis. *Helmet wearing* was non-significant (OR = 1.30, 95% CI: 0.88–1.91, p = 0.182) but below the nominal threshold for inclusion in the multivariable model. With the exception of *helmet wearing*, all terms in the initial multivariable logistic regression were significant and the re-inclusion of *bikelane* and *time of day* was not justified (p = 0.53 and p = 0.77 respectively). The variables *vehicle size*, *city* and *kerb distance* are significant in the final model while *helmet wearing* remained non-significant with a smaller adjusted effect (aOR = 1.13, 95% CI: 0.76–1.68, p = 0.54). Although highly non-significant, *helmet wearing* has been retained in the final multivariable model as it is the primary focus of this paper. This non-significance is evident in the raw data as far passing manoeuvres occurred 94.8% and 95.9% of the time when helmeted and unhelmeted respectively. It is also evident in the estimated proportions of unsafe passing distance by *kerb distance* and *helmet wearing* in Figure 2.

**Table 4:** Univariate and multiple logistic regression analysis for close passing (<1 m).

	Univariate			Multivariable		
	OR	95% CI	p- value	aOR	95% CI	p- value
Vehicle, Small vs. Large	0.53	0.34–0.81	0.004	0.58	0.38–0.90	0.016
Bikelane, Yes vs. No	0.86	0.21–3.57	0.831			
*City	0.61	0.39–0.93	0.023	0.46	0.28–0.77	0.003
Time of Day						
7–10 AM	1.43	0.86–2.36	0.368			
10–2 PM	1.35	0.87–2.12	0.533			
2 PM+	(referent)					
Kerb Distance						
0.25 m	0.21	0.11–0.40	<0.001	0.18	0.09–0.36	<0.001
0.50 m	0.44	0.25–0.78	0.005	0.30	0.16–0.58	<0.001
0.75 m	0.73	0.41–1.29	0.274	0.54	0.29–1.01	0.052
1.00 m	0.54	0.31–0.95	0.032	0.51	0.29–0.89	0.018
1.25 m	(referent)					
Helmet, Yes vs. No	1.30	0.88–1.91	0.182	1.13	0.76–1.68	0.540

* City comparison is between Salisbury and Bristol.

The final multiple logistic model was rerun for additional cut points for close passing distance defined by 0.5 m, 0.75 m, 1.5 m and 2.0 m. The results are given in Table 5. The adjusted odds ratio is near one using the 0.75 m cut point and increases with increasing cut points. The odds ratio is statistically significant for cut points of 1.5 m and 2.0 m.

**Table 5:** Estimates of helmet wearing effect (Yes vs. No) in multiple logistic regression analysis for close passing using various cut points.

Cut point	aOR	95% CI	p-value
0.5m*	–	–	–
0.75m^+^	1.01	0.33–3.11	0.993
1.0m	1.13	0.76–1.68	0.540
1.5m	1.21	1.02–1.44	0.028
2.0m	1.46	1.13–1.89	0.004

* There were only two passing events less than 0.5 m. In each case, Walker did not wear a helmet.

+There were only 13 overtaking events less than 0.75 m. Close passing events occurred 0.6% and 0.5% of the time when wearing and not wearing a helmet respectively.

Sample sizes for small, medium and large effect sizes and 80%, 85% and 90% power were computed separately for analysing passing distance as a continuous and categorical variable using G*Power. Bootstrap p-values from linear models with passing distance as the dependent variable are given in Table 6. *Helmet wearing* when adjusted for other variables is only statistically significant at the 5% level for sample sizes computed for small effect sizes. Conversely, *kerb distance* is highly significant in each of the nine power/effect size combinations. When passing distance is categorised as close passing (<1 m) (Table 7), *helmet wearing* is non-significant in each case except for univariate models with sample sizes computed from small effect sizes, while *kerb distance* is statistically significant for multivariable models in eight of the nine power/effect size combinations with the exception being marginally insignificant (p = 0.051).

**Table 6:** Bootstrap p-values from univariate (UV) and multivariable (MV+) linear models of helmet wearing and kerb distance on passing distance.

			Linear Regression			
			Helmet Wearing		Kerb Distance	
Power	Effect Size	Sample Size*	UV	MV^+^	UV	MV^+^
0.80	Small (*f = 0.1*)	1580	<0.001	0.003	<0.001	<0.001
	Medium (*f = 0.25*)	260	0.060	0.187	<0.001	<0.001
	Large (*f = 0.40*)	110	0.231	0.402	0.005	0.006
0.85	Small (*f = 0.1*)	1760	<0.001	0.001	<0.001	<0.001
	Medium (*f = 0.25*)	290	0.052	0.170	<0.001	<0.001
	Large (*f = 0.40*)	120	0.242	0.417	0.004	0.004
0.90	Small (*f = 0.1*)	2000	<0.001	<0.001	<0.001	<0.001
	Medium (*f = 0.25*)	330	0.048	0.168	<0.001	<0.001
	Large (*f = 0.40*)	140	0.214	0.407	0.001	0.002

* Sample sizes computed using G*Power 3.1.3 with α = 0.05 for a 2×5 factorial fixed effects.

+ Multivariable models include vehicle size, city, helmet wearing and kerb distance.

**Table 7:** Bootstrap p-values from univariate (UV) and multivariable (MV+) logistic regression models of helmet wearing and kerb distance on close overtaking (<1m).

			Helmet Wearing		Kerb Distance	
Power	Effect Size	Sample Size*	UV	MV^+^	UV	MV^+^
0.80	Small (*OR = 1.22*)	14023	0.001	0.130	<0.001	<0.001
	Medium (*OR = 1.86*)	1224	0.307	0.583	<0.001	<0.001
	Large (*OR = 3.00*)	335	0.750	0.928	0.072	0.051
0.85	Small (*OR = 1.22*)	16035	<0.001	0.085	<0.001	<0.001
	Medium (*OR = 1.86*)	1396	0.319	0.626	<0.001	<0.001
	Large (*OR = 3.00*)	381	0.642	0.828	0.063	0.039
0.90	Small (*OR = 1.22*)	18760	<0.001	0.081	<0.001	<0.001
	Medium (*OR = 1.86*)	1628	0.280	0.655	<0.001	<0.001
	Large (*OR = 3.00*)	441	0.600	0.794	0.053	0.031

* Sample sizes computed using G*Power 3.1.3 for a logistic regression with α = 0.05, probability of close overtaking while wearing a helmet of 0.055, and probability of wearing a helmet of 0.5.

+ Multivariable models include vehicle size, city and kerb distance.

## Discussion

In this manuscript, we set out to re-analyse the Walker data on motor vehicle passing distance while riding a bicycle. Additional univariate and multivariable analyses were undertaken to assess whether helmet wearing was associated with passing manoeuvres less than the recommended one metre and to assess the influence sample size had on the statistical significance of helmet wearing in the original study.

This re-analysis found significant associations not previously identified in the original study. There was a significant decline in the adjusted odds of close (<1 m) versus far passing manoeuvres for vehicle size (aOR = 0.58, 95% CI: 0.38–0.90), cycling in Salisbury (aOR = 0.46, 95% CI: 0.28–0.77) and distance to the kerb (0.25 m vs. 1.25 m, aOR = 0.18, 95% CI: 0.09–0.36). These factors were also associated with passing distance as a continuous measure.

Our analysis confirms Walker's results regarding helmets and passing distance with an adjusted estimate of an additional 5.8 cm when not wearing a helmet. However, the magnitude of this effect is a 32% decrease from the unadjusted estimate and was less than the estimated effects of vehicle size, city and kerb distance all having a larger impact on passing distance. Additionally, the odds ratio for helmet wearing and safe passing distance decreased with lower cut points suggesting differences in overtaking distance for helmeted versus unhelmeted events is only observed for the close passing cut point defined as 1.5 m or more. This becomes clear when average passing distances are computed for cut point intervals by helmet wearing (Table 8). Only distances greater than 2.0 m are statistically significant for helmet wearing and the magnitude of the difference is 7 cm. Given that any evidence of a difference in passing distance related to helmet wearing is only observed for passing distances well above the recommended one metre, these results do not support the idea that any substantive risk reduction can be gained from not wearing a helmet.

**Table 8:** Mean passing distance (in metres) by helmet wearing for intervals created using various cut points.

	Helmet		No Helmet		
Passing distance	mean	n	mean	n	p-value*
[0,0.75)	0.66	7	0.61	6	0.556
[0.75,1.0)	0.90	53	0.90	43	0.924
[1.0,1.5)	1.29	524	1.30	469	0.465
[1.5,2.0)	1.70	455	1.72	508	0.103
[2.0, ∞)	2.22	110	2.29	180	<0.001

* contrasts from 2×5 ANOVA.

The results using bootstrap standard error estimates further support the notion that the effect of helmet wearing is at most a minimal effect on passing distance. By contrast, kerb distance was highly significant under every condition, while helmet wearing was only significant under a more constrained set of conditions. This suggests kerb distance is an important factor in passing distance and helmet wearing is not. This is also clear when analysing only helmet wearing and kerb distance as in the original study. Walker [2] reported a coefficient of determination of 8% when helmet wearing and kerb distance are in the model. When separate models are estimated, it is clear that the majority of the variability accounted for in the model is due to kerb distance (r^2^ = 0.012 and r^2^ = 0.065 for helmet and kerb distance respectively).

Street type would appear to be an important factor as the street's width gives an upper bound for possible passing distance. However, there was no objective measurement of road width due to constant variation in unique road characteristics during each passing manoeuvre. Although non-significant in the univariate analysis for passing distance, *city*, as a proxy for the confounded variable *street type*, was significant when added back into the final multivariable model suggesting potential confounding with other variables. Since *city* was significant for both univariate and multivariable logistic models, it is unlikely its importance is an anomaly. The significant effect of *city* on passing distance and close/far passing does lend some credence to the hypothesis that street type influences passing distance. Further, using data from the 2010 midyear population estimates [40], [41], the population density of Bristol (4,012/km^2^) is much larger than Salisbury (1,612/km^2^) which suggests there may be less room for passing in highly populated areas. However, there may be other factors particular to the infrastructure, geography and road culture of each city that affect passing distance.

**Figure 3:**
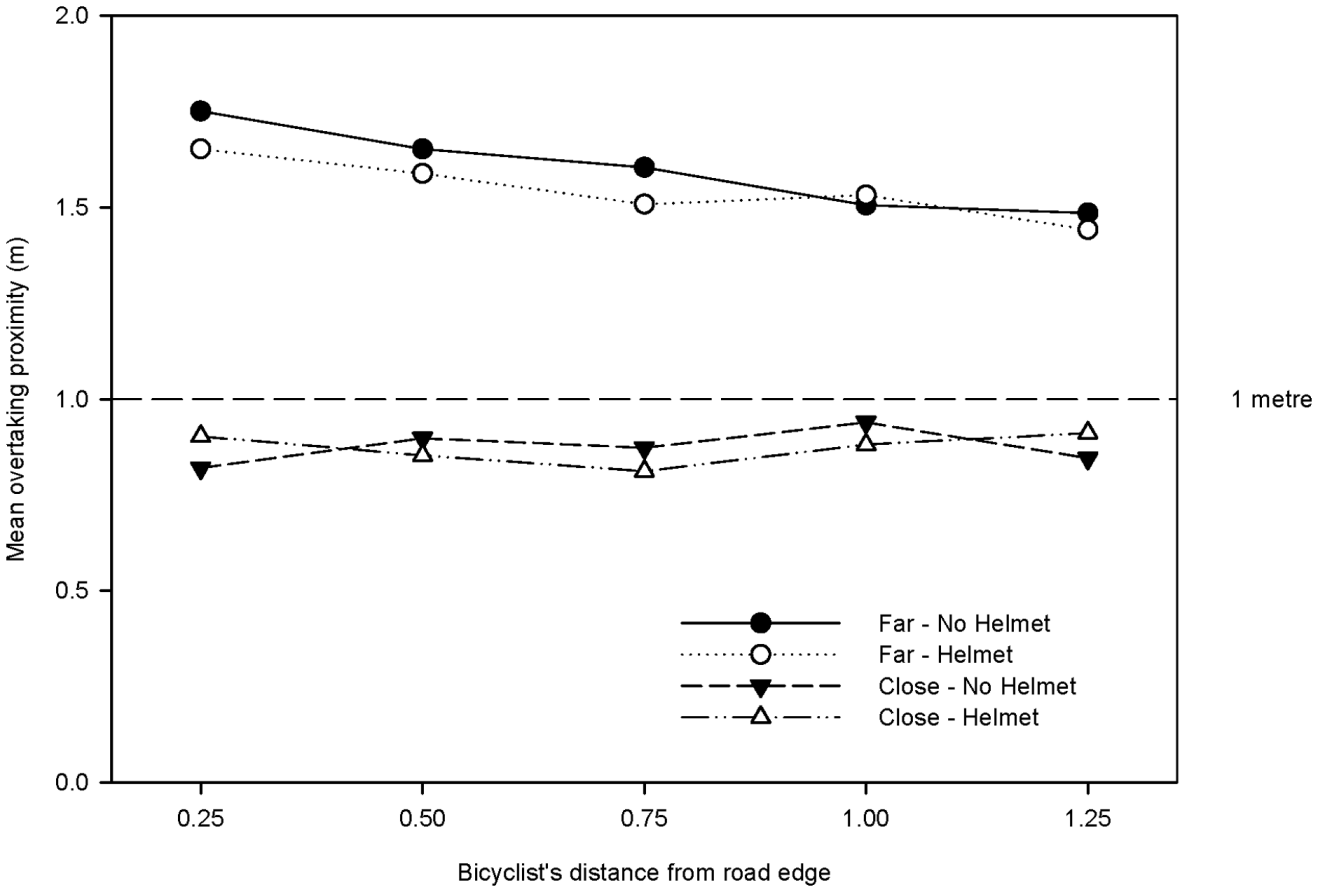
Mean overtaking distance for close/far passing manoeuvres, helmet wearing and bicyclist's distance from road edge.

When passing distance is categorised into close and far, the relationship with helmet wearing becomes inconsequential and is at odds with the results of the original paper. When taken at each fixed distance to the kerb, the estimated proportion of close passing manoeuvres is not systematically different when wearing and not wearing a helmet. Yet, there is a significant difference in passing distance overall related to helmet wearing. When passing distance is plotted versus kerb distance for close/far passing manoeuvres and helmet wearing categories (see Figure 3), it is clear the reported additional average passing space when not wearing a helmet occurs only during overtaking events of at least one metre. Therefore, with regards to bicycle safety, helmet wearing does not appear to influence unsafe driving behaviour.

Walker reported a significant odds ratio of 1.4 related to helmet wearing when comparing *near* and *far* passing manoeuvres, based on data-driven lower and upper quartiles, respectively. There were 590 *near* overtaking events based on this categorisation of which 481 (81.5%) were at distances of at least one metre. Because the vast majority of these events are considered safe by the one metre rule, it is possible the data driven categorisation has biased his reported results [25].

Walker importantly noted that when a cyclist moves away from the kerb, a car will pass with continually less passing distance. Analogously, we found that the odds of passing at less than a metre increases with greater kerb distance. As cycling increases in popularity, it is important to understand cyclist and driver interactions. Further research is needed in understanding these interactions in order to determine ways for cyclists and drivers to share a finite space so that risk of collision is minimised.

This study has several limitations with regards to the data. Ian Walker was the only cyclist observed and therefore his cycling behaviour may not be representative of cyclists in general. Additionally, the results may not be generalisable to jurisdictions with existing helmet laws where helmet wearing rates are much higher than the UK. For example, in a recent study in which 4225 cyclists in Melbourne, Australia were observed by video camera while at a red light, only eight were observed not wearing a helmet [42]. Thus, motor vehicle drivers would be more accustomed to helmeted cyclists in such locations.

## Conclusions

Risk compensation theory for helmet wearing while cycling has generated increased interest in the peer-reviewed literature, although there is little to no evidence to support the theory. Walker's [2] argument that helmet wearing affects the behaviour of motor vehicle drivers does not support risk compensation theory upon re-analysis. Helmet wearing is associated with a small difference in passing distance and is not associated with close passing. The evidence from this study does not justify recommendations around helmet wearing, but rather highlights the more important factors of kerb distance, road characteristics and traffic type which may inform more effective cycling safety improvements.
