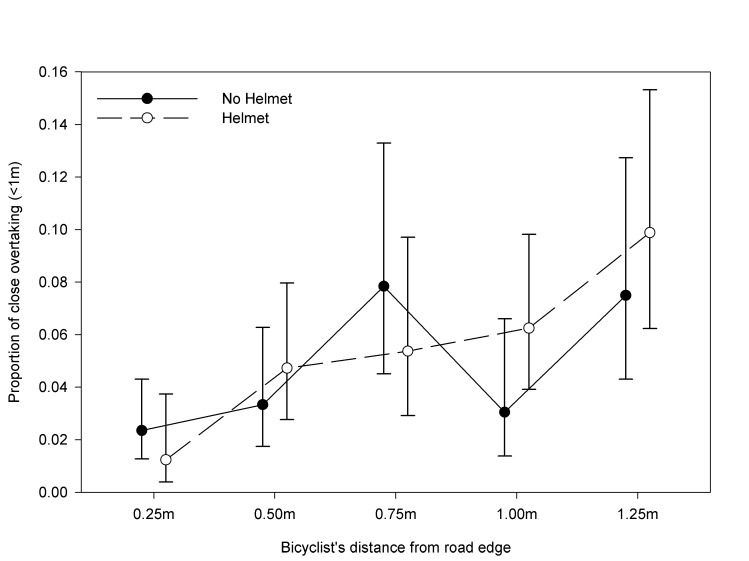# Correction: Bicycle Helmet Wearing Is Not Associated with Close Motor Vehicle Passing: A Re-Analysis of Walker, 2007

**DOI:** 10.1371/annotation/7e009550-a92d-49a2-8053-e6fcf7612966

**Published:** 2014-01-06

**Authors:** Jake Olivier, Scott R. Walter

In Figure 1 and Figure 2, the labels for "Helmet" and "No Helmet" were reversed. Please see the corrected Figure 1 here: 

**Figure:**
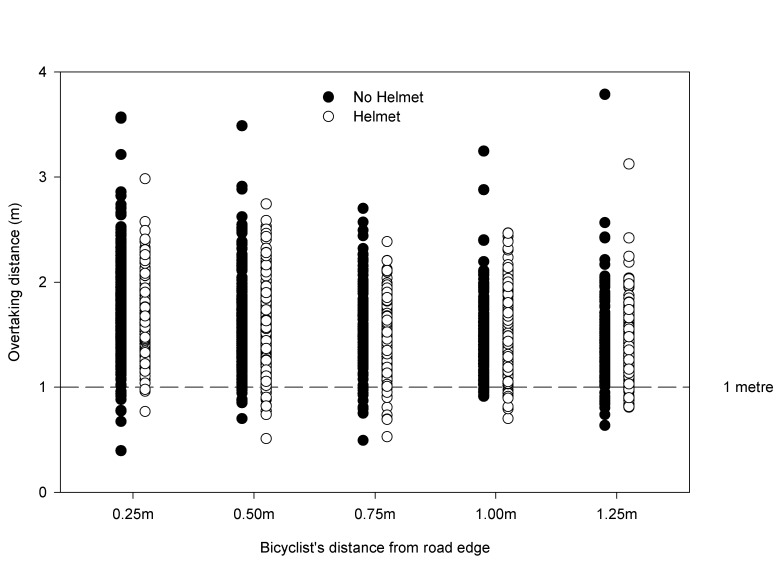


Please see the corrected Figure 2 here: 

**Figure:**